# RANKL Cytokine: From Pioneer of the Osteoimmunology Era to Cure for a Rare Disease

**DOI:** 10.1155/2013/412768

**Published:** 2013-05-15

**Authors:** Nadia Lo Iacono, Alessandra Pangrazio, Mario Abinun, Robbert Bredius, Marco Zecca, Harry C. Blair, Paolo Vezzoni, Anna Villa, Cristina Sobacchi

**Affiliations:** ^1^UOS/IRGB, Milan Unit, CNR, Milano, Italy; ^2^Humanitas Clinical and Research Center, Via Manzoni 113, 20089 Rozzano, Italy; ^3^Great North Children's Hospital, Institute for Cellular Medicine, Newcastle University, Newcastle upon Tyne, UK; ^4^Department of Pediatrics, Leiden University Medical Center, Leiden, The Netherlands; ^5^Pediatric Hematology/Oncology, Foundation IRCCS, San Matteo, Pavia, Italy; ^6^Department of Pathology, Veteran's Affairs Medical Center, University of Pittsburgh, Pittsburgh, PA, USA

## Abstract

Since its identification, the RANKL cytokine has been demonstrated to play a crucial role in bone homeostasis and lymphoid tissue organization. Genetic defects impairing its function lead to a peculiar form of autosomal recessive osteopetrosis (ARO), a rare genetic bone disease presenting early in life and characterized by increased bone density due to failure in bone resorption by the osteoclasts. Hematopoietic stem cell transplantation (HSCT) is the only option for the majority of patients affected by this life-threatening disease. However, the RANKL-dependent ARO does not gain any benefit from this approach, because the genetic defect is not intrinsic to the hematopoietic osteoclast lineage but rather to the mesenchymal one. Of note, we recently provided proof of concept of the efficacy of a pharmacological RANKL-based therapy to cure this form of the disease. Here we provide an overview of the diverse roles of RANKL in the bone and immune systems and review the clinical features of RANKL-deficient ARO patients and the results of our preclinical studies. We emphasize that these patients present a continuous worsening of the disease in the absence of a cure and strongly wish that the therapy we propose will be further developed.

## 1. Introduction

In accordance with the ancient Latin maxim, “*In medio stat virtus*”, in all the organisms the physiology of many biological functions is based on the equilibrium between the opposite activities of different cell types. In vertebrates, homeostasis of the skeletal tissue is accomplished by the balance between bone synthesis, performed by the osteoblasts, and bone resorption, carried out by the osteoclasts; in addition, other cell types, such as osteocytes and immune cells, as well as soluble factors, such as cytokines and hormones, cooperate to the same end [1]. Altering this balance leads to diseases characterized by either a decrease (as in osteoporosis) or increase (as in osteopetrosis) in bone mass. The term “osteopetrosis” (from the Ancient Greek *οστέον*, osteon = bone, + *πέτρος*, petros = stone) defines a number of monogenic disorders characterized by increased bone density due to failure in bone resorption by the osteoclasts, large multinucleated cells of hematopoietic origin. The most severe form is the autosomal recessive osteopetrosis (ARO), which presents soon after birth and is often lethal unless treated with hematopoietic stem cell transplantation (HSCT), providing the precursor cells for the differentiation of functional osteoclasts in the host [2–5]. Subgroups of patients with ARO are distinguished based on the affected gene [6]. Among them, the RANKL dependent is the most peculiar, since it bears defects in a gene mainly expressed, in bone, by mesenchymal-derived cells. Thus, HSCT is not a valid therapeutic option for this subset of patients [7]. Moreover, the discovery of RANKL has represented the beginning of a new scientific field, called “osteoimmunology”, tightly linking the bone to the immune system [8, 9]. Indeed, this cytokine is multitasking, with roles ranging from bone remodeling to lymphoid tissue organization [10, 11]. Its importance has recently been highlighted by the development of a monoclonal antibody against RANKL (denosumab, Amgen Inc., Thousand Oaks, CA, USA) approved in the clinical practice for the treatment of postmenopausal osteoporosis and cancer-related osteolysis [12] and under evaluation in a Phase 4 clinical trial for rheumatoid arthritis (clinical trial identifier: NCT01770106). Here we summarize the many roles of RANKL in the bone and immune systems, review the clinical features of RANKL-deficient ARO patients, and discuss the results of preclinical studies on a RANKL-based pharmacological therapy holding great promise for these patients.

## 2. RANKL in Bone

RANKL, also called TNF-related activation induced cytokine, TRANCE; osteoclast differentiation factor, ODF; and osteoprotegerin ligand, OPGL, is a type II transmembrane protein belonging to the TNF superfamily, whose gene was cloned fifteen years ago by four different groups contemporaneously [13–16]. It exists predominantly in a membrane-bound form, with a short cytoplasmic N-terminal domain and a single transmembrane region, but a soluble form can be generated through alternative splicing [17] or through the cleavage by matrix metalloproteinases and ADAMs (disintegrin and metalloproteinase domain-containing proteins) [18–20]. RANKL aggregates into homotrimers through conserved and specific residues in the extracellular domain, and trimerization is essential for the activation of its cognate receptor RANK [21–24]. Recently, the crystal structure of human RANKL in complex with its decoy receptor osteoprotegerin (OPG) has been determined, too, and showed that in this case a different mode of interaction takes place, directly blocking the accessibility of residues of RANKL important for RANK recognition [25, 26].

RANKL is broadly expressed, including both skeletal and extra-skeletal sites and many diverse cell types, such as T and B lymphocytes, mammary epithelial cells, keratinocytes, vascular endothelial cells, and synovial fibroblasts [15, 17, 27–29]. In the bone, it is produced mainly by cells of mesenchymal origin, osteoblasts, hypertrophic chondrocytes, and bone marrow (BM) stromal cells; recently, osteocytes have been identified as another source of this cytokine [30–32]. Its expression is positively regulated by several factors, such as parathyroid hormone (PTH) and 1,25 dihydroxyvitamin D_3_, which bind to a distal control region (DCR) located 76 kb upstream of the murine *Rankl* transcription start site [33]; deletion of the DCR in mouse significantly affects Rankl production and the rate of bone remodeling [34]. Other factors stimulating *Rankl* expression are calcium, glucocorticoids, prostaglandin E2, interleukin (IL)-1*α*, IL-6, IL-11, and IL-17, while the canonical Wnt signaling and transforming growth factor (TGF)-*β* pathways downregulate it [29, 35].

Together with M-CSF, RANKL is the master cytokine driving osteoclast differentiation through the binding to its receptor RANK and the activation of different intracellular signaling cascades, involving an increasing number of molecules; among them, TRAF6, NF-kB, ERK1/2, JNK, and p38 have ultimately, as target gene *NFATc1*, the crucial transcription factor in osteoclastogenesis [12, 36, 37]. The strongest evidence for the role of RANKL during osteoclastogenesis came from gene inactivation in murine models [38–40], leading to osteoclast-poor osteopetrosis already present at birth. At 1 month of age, *Rankl* ^−/−^ mice were severely growth retarded due to poor nutrition secondary to lack of tooth eruption and displayed shortened long bones with club-shaped ends, thinning of the calvariae, generalized increase in bone density with very little marrow space, marked chondrodysplasia with thick, irregular growth plates, and relative increase in hypertrophic chondrocytes. At an older age, *Rankl* ^−/−^ mice developed rounded faces, likely due to osteopetrotic changes of the facial skeleton. The phenotype was only partially rescued by transgenic overexpression under a lymphocyte-specific promoter, highlighting the importance of RANKL local delivery in many skeletal compartments [39, 40]. More recently, conditional knockout models have been generated by specifically targeting the gene in chondrocytes, at different stages of the osteoblast lineage or in osteocytes [41, 42]. The deletion of the gene in chondrocytes or in the osteoblasts led to severe osteopetrosis in mice at birth, while the deletion in the osteocytes caused a pathologic bone phenotype only later in postnatal life, suggesting a different contribution of these cell types in bone modeling and remodeling [31, 32, 43]. In addition, during the screening of N-ethyl-N-nitrosourea (ENU)-mutagenized mice, Douni and colleagues have recently reported a new mouse model bearing a G278R substitution in Rankl (*Rankl* ^*tles*/*tles*^); this mutation was predicted to impair trimerization, affecting the bone phenotype to the same extent as in the *Rankl* ^−/−^ mouse [44]. Other osteoclast differentiation pathways independent from the RANKL/RANK axis have been reported, such as those driven by TGF*β* or LIGHT [45–47]; however, the phenotype of the murine models above described, as well as the osteopetrotic features of *Rank* knockout mice [48–50], clearly indicates that *in vivo* those alternative pathways cannot completely substitute for a lack of signal from the RANKL/RANK system.

On the other hand, an over activity of this pathway has been described to contribute to conditions characterized by excessive bone loss or destruction such as osteoporosis, cancer-related osteolysis, and Paget's disease [51, 52], giving thus the *rationale* for the establishment of an anti-RANKL therapy in these patients.

## 3. RANKL in the Immune System

At the very beginning of its story, RANKL was described as a dendritic cell (DC) survival factor allowing efficient priming of T cells [13, 14]. Interestingly, this cell type did not appear to be affected in *Rankl* ^−/−^ mice, as far as it was investigated [38]. However, these mice displayed other clear immunological defects, even though with some differences likely due to the genetic background. Kong and colleagues reported reduced thymus size and cellularity, thymocyte development block at the stage CD4^−^CD8^−^CD44^−^CD25^+^, and defective cytokine production. They also found spleen enlargement and block in the progression of B220^+^CD25^−^ pro-B cells to B220^+^CD25^+^ pre-B cells, in the presence of intact splenic architecture, with normal distribution of red and white pulp, normal T- and B-cell segregation and normal primary follicle structure [38]. In an independent *Rankl* ^−/−^ model, Kim and colleagues described altered splenic microarchitecture and defects in B-cell follicle formation and in marginal zone integrity in the majority of *Rankl* ^−/−^ mice [53]. In the same model, red pulp expansion, white pulp reduction, and regions of intense extramedullary hematopoiesis in the spleen occur, together with severe reduction in thymic medulla [54]. Also in the *Rankl* ^*tles*/*tles*^ mouse, thymic hypoplasia and enlarged spleen have been reported [44]. Moreover, all these models displayed complete lack of lymph nodes (LNs; cervical LNs were seldom present) and smaller Peyer's patches [38, 44, 53].

These findings are consistent with the diverse functions RANKL exerts in the immune system: during LN organogenesis, together with LT*αβ*, it regulates the colonization of the forming anlagen by CD45^+^CD4^+^CD3^−^ cells [53]. In the thymus, it is required for autoimmune regulator (AIRE)-expressing medullary thymic epithelial cell maturation [55–58]. In the skin, after different environmental stimuli, it mediates immunosuppression by increasing regulatory T-cell numbers [59]. In the gut, it initiates the development of antigen-sampling M cells in the intestinal epithelium [60] and is essential for the CXCL13-dependent maturation of cryptopatches into isolated lymphoid follicles in the small intestine [61].

In addition, several reports have highlighted the cross-talk between immune and bone cells through this molecule, and the importance of the contribution of immune cells becomes particularly evident in pathological conditions. For example, Th1 and Th2 cells inhibit osteoclastogenesis through the production of IFN-*γ* and IL-4, while Th17 cells induce osteoclast formation and osteolysis in rheumatoid arthritis (RA) via the IL-17-mediated induction of RANKL expression on synovial fibroblasts [9, 62]. In addition, a role for B cells in the pathogenesis of RA has been suggested by the significant efficacy of the treatment with an anti-CD20 antibody in cases showing an inadequate response to anti-TNF therapies [63]. RANKL produced by B cells also contributes to bone resorption during periodontal infection [64, 65] and to the increase in osteoclasts and trabecular bone loss occurring upon estrogen withdrawal [66]. Based on these interconnections, the RANKL/RANK axis has rightly been defined an essential regulator of both immune responses and bone physiology [67], and it is largely expected that alterations in one system will also affect the other.

## 4. RANKL-Dependent ARO Patients: A Small Group of Great Interest

In 2007 our group described for the first time mutations in the *RANKL* gene in 6 patients from 4 families affected by ARO [7]; in this review we refer to these individuals using the same nomenclature. Subsequently, we identified 3 additional patients with mutations in *RANKL*; no detailed clinical data are available for 2 of them (referred to as S5 and S6); for the remaining one (referred to as S7), the entire clinical history is herein reported for the first time. The mutation details are reported in Table 1. No other reports on this ARO subset exist in the literature, to the best of our knowledge; therefore, at present RANKL-dependent ARO represents about 3% of all ARO forms in our cohort of about 300 patients.

In the original work, onset of the disease was reported to range from 2 days to 1 year of age; at diagnosis, patients presented with fractures (4 of 6), visual impairment (5 of 6; S2A, S2B, and S4 underwent bilateral optic nerve decompression, without benefit), neurological defects (hydrocephalus, nystagmus; 4 out of 6), hepatosplenomegaly (from minimal to important, in all of them), and lack of palpable lymph nodes but no overt immunological defects. Three of them received full HSCT before the molecular diagnosis (S1, S2A, and S3A); they showed good levels of hematological engraftment but no improvement in bone remodeling. This prompted us to hypothesize a role for an osteoclast-extrinsic factor in the pathogenesis of the disease in these individuals, as also suggested by the evidence of lack of osteoclasts in the bone biopsy specimen of 4 of them and by the ability to differentiate functional osteoclasts from the patients' PBMCs *in vitro*. Indeed, all of them bore homozygous mutations in *RANKL* gene: patient S1 carried a deletion of five nucleotides in intron 7 resulting in skipping of exon 7 and in-frame deletion encompassing amino acids (aa) 145–177. Patients S2A, S2B and S4 displayed a single nucleotide substitution in exon 8 causing a missense mutation; the same amino acid change was subsequently found in patients S5 and S6 [6]. Patients S3A and S3B bore a deletion of two nucleotides in exon 8 leading to a frameshift at the C-terminus of the protein [7]. Based on early crystallographic studies [21], these mutations were predicted to affect regions important for RANKL trimerization or for the interaction with RANK. The same mutations were further investigated subsequently by *in vitro* osteoclastogenesis assays using mutant constructs [36] and by crystallographic studies of the murine RANKL ectodomain in complex with the RANK ectodomain [24]. In their study, Crockett and colleagues could not draw definitive conclusions regarding the possibility that the mutant products, in particular the missense mutation, maintain a residual activity or rather completely lose their function [36]. On the other hand, Ta and colleagues confirmed that the deletion of aa 145–177 abolished the interaction with RANK and the frameshift affected conformation and binding activity; the missense mutation proved to be more difficult to analyze possibly due to protein instability or misfolding [24].

Regarding the immunological compartment, at the time of our first work, 3 patients had already received HSCT, therefore were not candidates to such studies; in the remaining ones, although exhaustive analyses were not possible due to difficulty in obtaining blood samples from the patients, we did not find differences with normal controls with regard to B- and T-cell subsets, T-cell proliferation and propensity to apoptosis; however, we detected lower levels of both Th1 and Th2 cytokines in one patient (S2B) after stimulation.

The recently identified patient S7 was born from consanguineous parents (first degree consanguinity) of Lebanese origin. The disease presented soon after birth with hypocalcemic seizures, increased bone density, several fractures, pancytopenia, failure to thrive, and cranial nerve involvement. She received a first HSCT from an HLA-matched family donor (healthy brother) at 2 years of age; lack of bone rescue raised the hypothesis of poor engraftment, so a second transplantation was performed 1 year later. In this case, full engraftment was documented by karyotype analysis and subsequently confirmed using DNA sequencing, but again there was no benefit on bone architecture. In spite of the early and severe clinical picture at presentation, the patient became a long-term survivor and was referred to our center for genetic investigation at 22 years of age. The analysis revealed the presence of a c.667C>T mutation in *RANKL*, leading to premature termination of translation (p.Arg223X), in the homozygous state; the same nucleotide change was found in the heterozygous state in her mother and in her healthy brother (the paternal DNA was not available for investigation). The possibility that a truncated protein might be produced from this mutated allele has not been verified; however, if synthesized, the predicted peptide would lack a large part of the extracellular domain and both homodimerization and receptor-binding functions are expected to be importantly impaired.

## 5. Followup of RANKL-Deficient Patients

The followup of the patients originally described and of patient S7 clearly show progressive worsening of clinical features in all of them.

At 11 years of age, 10 years after transplantation, patient S1 showed neurological deterioration due to increasing compression of cerebro-bulbar structures; afterwards she was lost to followup.

At 14 years of age, 5 years post-HSCT, patient S2A developed hemolytic anemia, requiring repeated transfusions and chelation. No adequate response to steroids, high-dose intravenous immunoglobulins (i.v. Ig), or rituximab therapy was achieved. At 16 years of age she displayed very severe growth retardation with both weight and height well below the 0.4th centile for age (height 109 cm, weight 26 kg), refractory to a trial with growth hormone. Bone defects included deformities, particularly affecting legs and knees and partially due to recurrent fractures, and severely impaired dentition. She also had an episode of osteomyelitis of her toe. At present, she is 18 years old with delayed puberty. She has recently been diagnosed with depression.

Her younger sister, patient S2B, at 10 years of age was severely growth retarded too (height 94.5 cm, weight 13.8 kg, both well below the 0.4th centile for age, and almost identical to her sister's growth pattern). Dentition was delayed, complicated by gum infection and more recently by chronic bilateral parotid and mandibular bone abscesses with sinusitis. She had experienced recurrent fractures at a younger age, which are properly healed. Like S2A, she developed anemia and became transfusion dependent at 11 years of age, soon requiring the use of chelating agents. Severe sleep apnea led to adenoidectomy; in addition, a reservoir was placed to resolve hydrocephalus several years ago. At present, she is 13 years old, has nocturnal continuous positive airway pressure (CPAP), and is complaining of recurrent and severe pounding headaches.

At 19 years of age, 11 years post-HSCT, patient S3A shows multiple poorly healing, traumatic fractures of her lumbar spine and progressive kyphosis of her neck secondary to lack of support by muscles. She is extremely growth retarded (height 118.5 cm, weight 27 kg, and both well below the 0.4th centile for age) with delayed puberty and defective dentition. She is anemic (Hb 5.5 mmol/L; normal range 7.5–10.0 mmol/L) and thrombocytopenic (123 × 10^9^/L; normal range 150–400 × 10^9^/L) but does not require transfusions.

At 4.5 years of age, her younger cousin, patient S3B displayed several neurological problems, including facial nerve palsy, headache, deterioration of hearing, due to progressive narrowing of the posterior fossa, and foramen magnum, as demonstrated by CT scan. The insertion of a ventriculoperitoneal (VP) shunt was required. At present, she is 6 years old, with severe growth retardation (height 94.6 cm, weight 13.6 kg, and both well below the 0.4th centile for age), has abnormal delayed dentition, poorly healing bone fractures, and recurrent ear and upper airway infections.

Patient S4 experienced multiple fractures which are, however, properly healed. Upper airway obstruction and hypoxic encephalopathy led to tracheostomy. At present, he is 6.5 years old and shows moderate growth retardation (height 105 cm, weight 16 kg, and both on the 4th centile) and markedly delayed tooth eruption (only 2 in lower jaws). He has episodes of severe agonizing headaches; CT and MRI brain scans showed a crowded posterior fossa and Chiari I malformation, but there were no signs of raised intracranial pressure. He has also recently had an episode of inflammatory synovitis of his left ankle.

**Figure 1:**
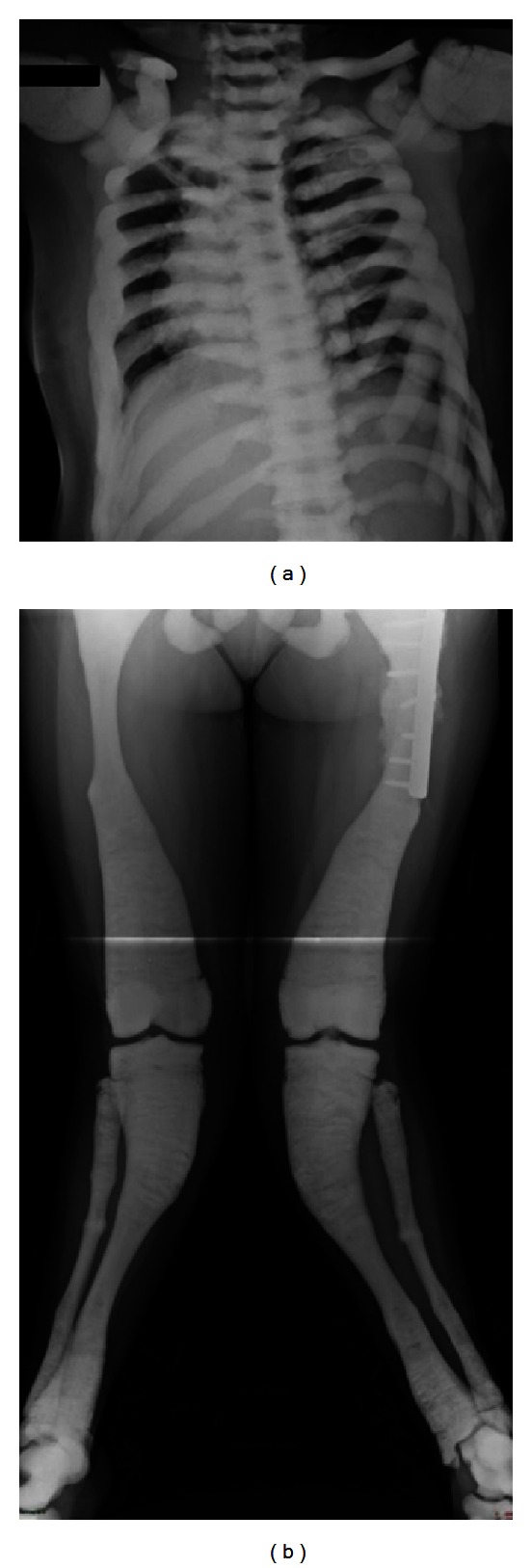
X-rays of patient S7 at the last followup showing right scoliotic deviation of the dorsal spine, knee valgus, and deviation of the tibia with multiple fractures of the tibia and fibula.

Patient S7 is now 22 years old, 18 years after the second transplant, and shows very severe growth retardation similarly to the other patients (height 120 cm, weight 38 kg, and both well below the 0.4th centile for age). Severe bone deformities involve all skeletal segments, with tendency to turricephaly, beak-like nose, exophthalmos, hypoplasia of the facial, and a more profound involvement of the spine, knees and legs (Figure 1). In the past years, she had very frequent bone fractures, in some cases with poor healing, requiring surgical intervention. Dental eruption is largely incomplete. The girl is totally blind and unable to articulate complete sentences, even though she can hear properly. Psychomotorial development is severely retarded. Episodes of agitation have been reported, with outbursts of anger and violence towards others. Nevertheless an MRI scan performed at the last control showed diffuse thickening of all the bones of the skull but a very mild cerebral atrophy with absence of other signs of central nervous system degeneration or deterioration. In addition, she suffers from recurrent pulmonary and upper airway infections.

Overall, these data demonstrate the need for the development of targeted approaches which could at least improve the quality of life of these patients.

## 6. A Pharmacological Therapy for RANKL-Dependent ARO: Preclinical Data Hold Promise

Since the identification of RANKL as the essential factor required for the osteoclastogenic process, exogenous administration of the soluble cytokine to murine and rat models have shown a great impact on bone metabolism and structure [15, 68–72].

In 1998, Lacey and colleagues tested the response of wild-type mice to a 3-day treatment with murine recombinant RANKL (aa 158–316) injected subcutaneously at different doses (1, 5, 25, and 100 *μ*g/day split in two administrations) [15]. They showed a rapid, significant, and dose-dependent increase in blood-ionized calcium level, a reduction in bone volume, and an increase in osteoclast cell size, with no change in osteoclast number. Soon after, the same group verified the effect of RANKL administration through a different route, that is, intravenously; doses ranged from 0.01 to 0.5 mg/kg, and mice were maintained either on a classic diet or on a low-calcium diet for 48 hours, in order to rule out the influence of gut calcium absorption [68]. They showed that the level of whole blood-ionized calcium increased dose-dependently after 1 hour and concluded that the observed effect was due to the activation of preexisting osteoclasts. In addition, they evaluated the role of RANKL in osteoclast survival through the subcutaneous injection of either saline or RANKL (1 mg/kg/day) in wild-type mice for 7 days, followed by a single OPG dose (10 mg/kg) [69]. They observed a two-fold increase in osteoclast number in RANKL-treated mice, while loss of stimulation by OPG administration rapidly led to osteoclast disappearance, thus pointing to an important role for RANKL in osteoclast survival.

In 2008, Lloyd and colleagues observed that bone turnover was greatly accelerated by the administration of soluble RANKL to wild-type mice (human recombinant RANKL, aa 143–317; dose 0.4 or 2 mg/kg/day split in two subcutaneous injections, for 10 days), with deleterious effects on both cortical and trabecular bone [70], in agreement with a possible role of this cytokine in the etiology of conditions characterized by pathological bone loss. The same group reported similar results after continuous RANKL infusion in rats for 28 days (2 doses: 35 *μ*g/kg/day or 175 *μ*g/kg/day), supporting also the appropriateness of this model for the study of high-turnover bone diseases [71]. In the same line, Tomimori and colleagues developed a model of rapid bone loss based on intraperitoneal daily injections of RANKL (a fusion protein of glutathione S-transferase (GST) and the extracellular domain of human RANKL, aa 140–317; dosages 0.5, 1, and 2 mg/kg) in wild-type mice, showing a dose-dependent decrease in bone mineral density (BMD) within 50 hours [72].

**Figure 2:**
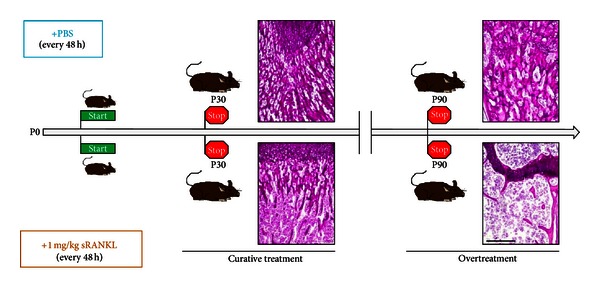
Preclinical studies in *Rankl* ^−/−^ mice. *Rankl* ^−/−^ mice were injected with either 1 mg/kg RANKL (below) or PBS (above) every 48 hours starting within the first week of life. Mice were sacrificed after 1 month (P30) or after 3 months (P90). Periodic acid-Schiff staining (bone in pink, cartilage in violet) of femur sections showed in RANKL-treated mice rescue of the bone defect at P30 and pathological reduction of the bone content at P90, clearly indicating overtreatment. Scale bar: 200 *μ*m.

Based on these *in vivo* evidences, when the human RANKL-dependent ARO subgroup was first identified [7], it was rather obvious to suggest that a pharmacological RANKL-based approach might be considered for the therapy of these patients. To test this hypothesis, we conducted a preclinical study treating *Rankl* ^−/−^ mice with subcutaneous injections of RANKL (soluble recombinant murine RANKL, amino acids 158–316, kindly provided by Amgen, Inc., Thousand Oaks, CA, USA; dosages 0.5, 1, or 2 mg/kg) from the first week of life, every 48 hours, for 1 month [54]. At sacrifice, we performed histological analysis of bone and all the main organs, including spleen, thymus, lung, heart, liver, kidney, pancreas, and mammary gland. The 1 mg/kg dose proved to be able to almost completely rescue the bone defect, by restoring osteoclast differentiation and resorption (Figure 2). Douni and colleagues reported a similar result in the bone compartment in their *Rankl* ^*tles*/*tles*^ mouse model, through the daily subcutaneous injection of RANKL (GST-fusion protein of murine RANKL, residues 158–316, dose 150 *μ*g/kg) from 13 days of age for 2 weeks [44]. Besides bone rescue, we also demonstrated that our protocol at the 1 mg/kg dose importantly ameliorated the hematolymphoid compartment of the treated *Rankl* ^−/−^ mice, with a restoration of the hematopoietic function within the bone marrow and an improvement of splenic and thymic architecture. Of note, no adverse effect was detected with this treatment regimen over the 1-month period of followup [54].

## 7. A Step Further Towards Patients

Our preclinical studies in *Rankl* ^−/−^ mice provided an important proof of concept of the efficacy of a RANKL-based pharmacological therapy. Further investigation is required to precisely identify all the possible toxic events related to RANKL administration. Our preliminary studies revealed detrimental effects associated with a clear overtreatment [53]; however, it should be considered that, when transferred to patients, many readouts of this therapy, such as serum calcium levels, concentration of a crosslink peptide sequence of type I collagen (CTX) in blood or urine, bone density by radiographic analysis, and immunophenotype, are easily available and indicative of the impact on bone metabolism, the immune system, and other physiological functions. Once a satisfactory bone response is obtained, the treatment regimen could be modified, for example, by dose tapering or periodical discontinuation, before adverse events arise.

As shown by the clinical data reported above, the RANKL-dependent ARO patients in our cohort, now in their teens in many cases, are in poor conditions, with some irreversible deficits (i.e. blindness) already established. We expect that the therapy we propose will stop further degeneration of the many impaired biological functions and will ameliorate their quality of life by reestablishing osteoclast formation and resorptive activity and improving the hematopoietic compartment. A major benefit is expected in the younger patients (S3B and S4).

The recent identification of a new RANKL-dependent ARO patient (S7) suggests that, while this subtype of the disease has been considered extremely rare, its frequency might be higher than expected: in fact, other patients, transplanted in the nineties before the characterization of the genetic basis of human osteopetrosis, might carry mutations in the *RANKL* gene. If they are still alive, they could be candidate to this new therapy, too.

The RANKL cytokine has been a pioneering discovery in the field of osteoimmunology; the elucidation of its signaling pathway has shown the first of the many, and continuously increasing, interconnections between the bone and immune systems. In the last years, a great interest has been deserved to therapies aimed at blocking this pathway and designed for diseases with increased bone resorption. On the other hand, the recognition of direct RANKL involvement in a genetic rare disease can constitute one of the few cases in which the result of a genetic study could also be translated into a replacement therapy. It is desirable that efforts from different entities, including research centers, clinics, charities, and biotech industries, might be joined in order to overcome the safety and regulatory issues and ultimately to give these patients not only a hope but a cure.

## Figures and Tables

**Table 1:** Molecular findings in RANKL-dependent ARO patients.

Patient	Genomic change^a^	cDNA change^b^	Protein change/effect^c^
S1	g.38250_38253delAGCT g.38250_38253delAGCT	c.532+4_532+8delAGCT c.532+4_532+8delAGCT	r.434_532del r.434_532del
S2A and S2B	g.43825T>A g.43825T>A	c.596T>A c.596T>A	p.Met199Lys p.Met199Lys
S3A and S3B	g.44057_44058delCG g.44057_44058delCG	c.828_829delCG c.828_829delCG	p.Val277TrpfsX5 p.Val277TrpfsX5
S4	g.43825T>A g.43825T>A	c.596T>A c.596T>A	p.Met199Lys p.Met199Lys
S5	g.43825T>A g.43825T>A	c.596T>A c.596T>A	p.Met199Lys p.Met199Lys
S6	g.43825T>A g.43825T>A	c.596T>A c.596T>A	p.Met199Lys p.Met199Lys
S7	g.43896C>T g.43896C>T	c.667C>T c.667C>T	p.Arg223X p.Arg223X

^a^Accession number genomic sequence of the *RANKL* gene: NG_008990.1.

^b^Accession number of the *RANKL* transcript variant 1: NM_003701.3; the numbering used starts with nucleotide +1 for the A of the ATG-translation initiation codon.

^c^Accession number of the RANKL protein isoform 1: NP_003692.1.

## References

[1] Sims NA, Walsh NC (2012). Intercellular cross-talk among bone cells: new factors and pathways. *Current Osteoporosis Reports*.

[2] Coccia PF, Krivit W, Cervenka J (1980). Successful bone-marrow transplantation for infantile malignant osteopetrosis. *The New England Journal of Medicine*.

[3] Gerritsen EJ, Vossen JM, Fasth A (1994). Bone marrow transplantation for autosomal recessive osteopetrosis. A report from the working party on inborn errors of the european bone marrow transplantation group. *Journal of Pediatrics*.

[4] Driessen GJA, Gerritsen EJA, Fischer A (2003). Long-term outcome of haematopoietic stem cell transplantation in autosomal recessive osteopetrosis: an EBMT report. *Bone Marrow Transplantation*.

[5] Mazzolari E, Forino C, Razza A, Porta F, Villa A, Notarangelo LD (2009). A single-center experience in 20 patients with infantile malignant osteopetrosis. *The American Journal of Hematology*.

[6] Villa A, Guerrini MM, Cassani B, Pangrazio A, Sobacchi C (2009). Infantile malignant, autosomal recessive osteopetrosis: the rich and the poor. *Calcified Tissue International*.

[7] Sobacchi C, Frattini A, Guerrini MM (2007). Osteoclast-poor human osteopetrosis due to mutations in the gene encoding RANKL. *Nature Genetics*.

[8] Rho J, Takami M, Choi Y (2004). Osteoimmunology: interactions of the immune and skeletal systems. *Molecular Cell*.

[9] Takayanagi H (2012). New developments in osteoimmunology. *Nature Reviews Rheumatology*.

[10] Hanada R, Leibbrandt A, Hanada T (2009). Central control of fever and female body temperature by RANKL/RANK. *Nature*.

[11] Hanada R, Hanada T, Sigl V, Schramek D, Penninger JM (2011). RANKL/RANK-beyond bones. *Journal of Molecular Medicine*.

[12] Lacey DL, Boyle WJ, Simonet WS (2012). Bench to bedside: elucidation of the OPG-RANK-RANKL pathway and the development of denosumab. *Nature Reviews Drug Discovery*.

[13] Anderson DM, Maraskovsky E, Billingsley WL (1997). A homologue of the TNF receptor and its ligand enhance T-cell growth and dendritic-cell function. *Nature*.

[14] Wong BR, Rho J, Arron J (1997). TRANCE is a novel ligand of the tumor necrosis factor receptor family that activates c-Jun N-terminal kinase in T cells. *The Journal of Biological Chemistry*.

[15] Lacey DL, Timms E, Tan HL (1998). Osteoprotegerin ligand is a cytokine that regulates osteoclast differentiation and activation. *Cell*.

[16] Yasuda H, Shima N, Nakagawa N (1998). Osteoclast differentiation factor is a ligand for osteoprotegerin/osteoclastogenesis-inhibitory factor and is identical to TRANCE/RANKL. *Proceedings of the National Academy of Sciences of the United States of America*.

[17] Ikeda T, Kasai M, Utsuyama M, Hirokawa K (2001). Determination of three isoforms of the receptor activator of nuclear factor-*κ*B ligand and their differential expression in bone and thymus. *Endocrinology*.

[18] Lum L, Wong BR, Josien R (1999). Evidence for a role of a tumor necrosis factor-*α* (TNF-*α*)-converting enzyme-like protease in shedding of TRANCE, a TNF family member involved in osteoclastogenesis and dendritic cell survival. *The Journal of Biological Chemistry*.

[19] Hikita A, Tanaka S (2007). Ectodomain shedding of receptor activator of NF-*κ*B ligand. *Advances in Experimental Medicine and Biology*.

[20] Hikita A, Tanaka N, Yamane S (2009). Involvement of a disintegrin and metalloproteinase 10 and 17 in shedding of tumor necrosis factor-*α*. *Biochemistry and Cell Biology*.

[21] Lam J, Nelson CA, Ross FP, Teitelbaum SL, Fremont DH (2001). Crystal structure of the TRANCE/RANKL cytokine reveals determinants of receptor-ligand specificity. *Journal of Clinical Investigation*.

[22] Ito S, Wakabayashi K, Ubukata O, Hayashi S, Okada F, Hata T (2002). Crystal structure of the extracellular domain of mouse RANK ligand at 2.2-Å resolution. *The Journal of Biological Chemistry*.

[23] Liu C, Walter TS, Huang P (2010). Structural and functional insights of RANKL-RANK interaction and signaling. *Journal of Immunology*.

[24] Ta HM, Nguyen GTT, Jin HM (2010). Structure-based development of a receptor activator of nuclear factor-*κ*B ligand (RANKL) inhibitor peptide and molecular basis for osteopetrosis. *Proceedings of the National Academy of Sciences of the United States of America*.

[25] Luan X, Lu Q, Jiang Y (2012). Crystal structure of human RANKL complexed with its decoy receptor osteoprotegerin. *Journal of Immunology*.

[26] Nelson CA, Warren JT, Wang MW, Teitelbaum SL, Fremont DH (2012). RANKL employs distinct binding modes to engage RANK and the osteoprotegerin decoy receptor. *Structure*.

[27] Kartsogiannis V, Zhou H, Horwood NJ (1999). Localization of RANKL (receptor activator of NF*κ*B ligand) mRNA and protein in skeletal and extraskeletal tissues. *Bone*.

[28] Nakashima T, Kobayashi Y, Yamasaki S (2000). Protein expression and functional difference of membrane-bound and soluble receptor activator of NF-*κ*B ligand: modulation of the expression by osteotropic factors and cytokines. *Biochemical and Biophysical Research Communications*.

[29] O’Brien CA (2010). Control of RANKL gene expression. *Bone*.

[30] Atkins GJ, Findlay DM (2012). Osteocyte regulation of bone mineral: a little give and take. *Osteoporosis International*.

[31] O'Brien CA, Nakashima T, Takayanagi H (2013). Osteocyte control of osteoclastogenesis. *Bone*.

[32] Xiong J, O'Brien CA (2012). Osteocyte RANKL: new insights into the control of bone remodeling. *Journal of Bone and Mineral Research*.

[33] Fu Q, Manolagas SC, O’Brien CA (2006). Parathyroid hormone controls receptor activator of NF-*κ*B ligand gene expression via a distant transcriptional enhancer. *Molecular and Cellular Biology*.

[34] Galli C, Zella LA, Fretz JA (2008). Targeted deletion of a distant transcriptional enhancer of the receptor activator of nuclear factor-*κ*B ligand gene reduces bone remodeling and increases bone mass. *Endocrinology*.

[35] Takahashi N, Maeda K, Ishihara A, Uehara S, Kobayashi Y (2011). Regulatory mechanism of osteoclastogenesis by RANKL and Wnt signals. *Frontiers in Bioscience*.

[36] Crockett JC, Mellis DJ, Scott DI, Helfrich MH (2011). New knowledge on critical osteoclast formation and activation pathways from study of rare genetic diseases of osteoclasts: focus on the RANK/RANKL axis. *Osteoporosis International*.

[37] Mellis DJ, Itzstein C, Helfrich MH, Crockett JC (2011). The skeleton: a multi-functional complex organ: the role of key signalling pathways in osteoclast differentiation and in bone resorption. *Journal of Endocrinology*.

[38] Kong YY, Yoshida H, Sarosi I (1999). OPGL is a key regulator of osteoclastogenesis, lymphocyte development and lymph-node organogenesis. *Nature*.

[39] Kim N, Odgren PR, Kim DK, Marks SC, Choi Y (2000). Diverse roles of the tumor necrosis factor family member TRANCE in skeletal physiology revealed by TRANCE deficiency and partial rescue by a lymphocyte-expressed TRANCE transgene. *Proceedings of the National Academy of Sciences of the United States of America*.

[40] Odgren PR, Kim N, MacKay CA, Mason-Savas A, Choi Y, Marks SC (2003). The role of RANKL (TRANCE/TNFSF11), a tumor necrosis factor family member, in skeletal development: effects of gene knockout and transgenic rescue. *Connective Tissue Research*.

[41] Xiong J, Onal M, Jilka RL, Weinstein RS, Manolagas SC, O'Brien CA (2011). Matrix-embedded cells control osteoclast formation. *Nature Medicine*.

[42] Nakashima T, Hayashi M, Fukunaga T (2011). Evidence for osteocyte regulation of bone homeostasis through RANKL expression. *Nature Medicine*.

[43] Nakashima T, Takayanagi H (2011). New regulation mechanisms of osteoclast differentiation. *Annals of the New York Academy of Sciences*.

[44] Douni E, Rinotas V, Makrinou E (2012). A RANKL G278R mutation causing osteopetrosis identifies a functional amino acid essential for trimer assembly in RANKL and TNF. *Human Molecular Genetics*.

[45] Itonaga I, Sabokbar A, Sun SG (2004). Transforming growth factor-*β* induces osteoclast formation in the absence of RANKL. *Bone*.

[46] Kim N, Kadono Y, Takami M (2005). Osteoclast differentiation independent of the TRANCE-RANK-TRAF6 axis. *Journal of Experimental Medicine*.

[47] Hemingway F, Kashima TG, Knowles HJ, Athanasou NA (2013). Investigation of osteoclastogenic signalling of the RANKL substitute LIGHT. *Experimental Molecular Pathology*.

[48] Dougall WC, Glaccum M, Charrier K (1999). RANK is essential for osteoclast and lymph node development. *Genes and Development*.

[49] Li J, Sarosi I, Yan XQ (2000). RANK is the intrinsic hematopoietic cell surface receptor that controls osteoclastogenesis and regulation of bone mass and calcium metabolism. *Proceedings of the National Academy of Sciences of the United States of America*.

[50] Kapur RP, Yao Z, Iida MHK (2004). Malignant autosomal recessive osteopetrosis caused by spontaneous mutation of murine Rank. *Journal of Bone and Mineral Research*.

[51] Goessl C, Katz L, Dougall WC (2012). The development of denosumab for the treatment of diseases of bone loss and cancer-induced bone destruction. *Annals of the New York Academy Science*.

[52] Chung PY, van Hul W (2012). Paget's disease of bone: evidence for complex pathogenetic interactions. *Seminars in Arthritis and Rheumatism*.

[53] Kim D, Mebius RE, MacMicking JD (2000). Regulation of peripheral lymph node genesis by the tumor necrosis factor family member TRANCE. *Journal of Experimental Medicine*.

[54] Lo Iacono N, Blair HC, Poliani PL (2012). Osteopetrosis rescue upon RANKL administration to Rankl(-/-) mice: a new therapy for human RANKL-dependent ARO. *Journal of Bone and Mineral Research*.

[55] Rossi SW, Kim MY, Leibbrandt A (2007). RANK signals from CD4+3- inducer cells regulate development of Aire-expressing epithelial cells in the thymic medulla. *Journal of Experimental Medicine*.

[56] Akiyama T, Shimo Y, Yanai H (2008). The tumor necrosis factor family receptors RANK and CD40 cooperatively establish the thymic medullary microenvironment and self-tolerance. *Immunity*.

[57] Hikosaka Y, Nitta T, Ohigashi I (2008). The cytokine RANKL produced by positively selected thymocytes fosters medullary thymic epithelial cells that express autoimmune regulator. *Immunity*.

[58] Desanti GE, Cowan JE, Baik S (2012). Developmentally regulated availability of RANKL and CD40 ligand reveals distinct mechanisms of fetal and adult cross-talk in the thymus medulla. *Journal of Immunology*.

[59] Loser K, Mehling A, Loeser S (2006). Epidermal RANKL controls regulatory T-cell numbers via activation of dendritic cells. *Nature Medicine*.

[60] Knoop KA, Kumar N, Butler BR (2009). RANKL is necessary and sufficient to initiate development of antigen-sampling M cells in the intestinal epithelium. *Journal of Immunology*.

[61] Knoop KA, Butler BR, Kumar N, Newberry RD, Williams IR (2011). Distinct developmental requirements for isolated lymphoid follicle formation in the small and large intestine: RANKL is essential only in the small intestine. *The American Journal of Pathology*.

[62] Kikuta J, Wada Y, Kowada T (2013). Dynamic visualization of RANKL and Th17-mediated osteoclast function. *Journal of Clinical Investigations*.

[63] Chan AC, Carter PJ (2012). Therapeutic antibodies for autoimmunity and inflammation. *Nature Reviews Immunology*.

[64] Han X, Kawai T, Eastcott JW, Taubman MA (2006). Bacterial-responsive B lymphocytes induce periodontal bone resorption. *Journal of Immunology*.

[65] Han X, Lin X, Seliger AR, Eastcott J, Kawai T, Taubman MA (2009). Expression of receptor activator of nuclear factor-*κ*B ligand by B cells in response to oral bacteria. *Oral Microbiology and Immunology*.

[66] Onal M, Xiong J, Chen X (2012). RANKL expression by B lymphocytes contributes to ovariectomy-induced bone loss. *The Journal of Biological Chemistry*.

[67] Leibbrandt A, Penninger JM (2008). RANK/RANKL: regulators of immune responses and bone physiology. *Annals of the New York Academy of Sciences*.

[68] Burgess TL, Qian YX, Kaufman S (1999). The ligand for osteoprotegerin (OPGL) directly activates mature osteoclasts. *Journal of Cell Biology*.

[69] Lacey DL, Tan HL, Lu J (2000). Osteoprotegerin ligand modulates murine osteoclast survival in vitro and in vivo. *The American Journal of Pathology*.

[70] Lloyd SAJ, Yuan YY, Kostenuik PJ (2008). Soluble RANKL induces high bone turnover and decreases bone volume, density, and strength in mice. *Calcified Tissue International*.

[71] Yuan YY, Kostenuik PJ, Ominsky MS (2008). Skeletal deterioration induced by RANKL infusion: a model for high-turnover bone disease. *Osteoporosis International*.

[72] Tomimori Y, Mori K, Koide M (2009). Evaluation of pharmaceuticals with a novel 50-hour animal model of bone loss. *Journal of Bone and Mineral Research*.

